# Long non-coding RNA TUG1 promotes colorectal cancer metastasis via EMT pathway

**DOI:** 10.18632/oncotarget.10563

**Published:** 2016-07-13

**Authors:** Liang Wang, Zhenxian Zhao, Weidong Feng, Zhijun Ye, Weigang Dai, Changhua Zhang, Jianjun Peng, Kaiming Wu

**Affiliations:** ^1^ Department of Gastrointestinal Surgery, First Affiliated Hospital, Sun Yat-sen University, Guangzhou, China; ^2^ Department of Biliary Pancreatic Surgery, First Affiliated Hospital, Sun Yat-sen University, Guangzhou, China

**Keywords:** long noncoding RNA, TUG1, CRC, EMT

## Abstract

Colorectal cancer (CRC) is the third most common malignancy in developed countries, and its incidence rate has been continuously increasing in developing countries over the past few decades. Taurine-upregulated gene 1 (TUG1) plays an important role in signal transduction, regulation of cell morphology, migration, proliferation and apoptosis. The aim of the present study was to evaluate the role of TUG1 in CRC, and whether knockdown of TUG1 expression could affect cell proliferation, migration and invasion of CRC cell lines. Here, we reported that TUG1 was upregulated in CRC. Further experiments revealed that TUG1 knockdown significantly inhibited cell proliferation, migration and invasion of CRC *in vitro*. Above all, knockdown of TUG1 may represent a rational therapeutic strategy for CRC patients in future.

## INTRODUCTION

Colorectal Cancer (CRC) is a lethal malignancy that threatens world-wide health [1]. CRC patients with early stage could be cured by surgery [2, 3]. Unfortunately, colectomy or rectectomy and chemotherapy are not appropriate for patients with advanced CRC [4]. The accumulation of genetic alterations mediates CRC progression by deregulating key signaling pathways in cancer cells. Thus, further understanding of molecular mechanisms of CRC may help develop novel therapeutic targets.

Long non-coding RNAs (lncRNAs), composed of more than 200 nucleotides, belong to non-coding RNAs (ncRNAs), with limited or no protein-coding capacity [5–7]. Previous studies have demonstrated that lncRNAs have exerted biological functions in cancer by participating in both oncogenic and tumor suppressing pathways [8–9]. LncRNAs also function as a competing endogenous RNA (ceRNA) and sponge miRNAs, regulating the expression of target mRNA.

Taurine-upregulated gene 1 (TUG1) was firstly reported to be upregulated in exposure to the treatment of taurine in mouse retinal cells [10]. It is reported that TUG1 overexpression was related to cell proliferation of various cancer [12–13]. However, the function of lncRNA TUG1 and its potential mechanism is not well illustrated in CRC. Here, we detected TUG1 expression in CRC tumor tissues and corresponding adjacent normal mucosa tissues. What's more, we also evaluate CRC cell proliferation, apoptosis, migration, and invasion *in vitro* after TUG1 knockdown.

## RESULTS

**Figure 1:**
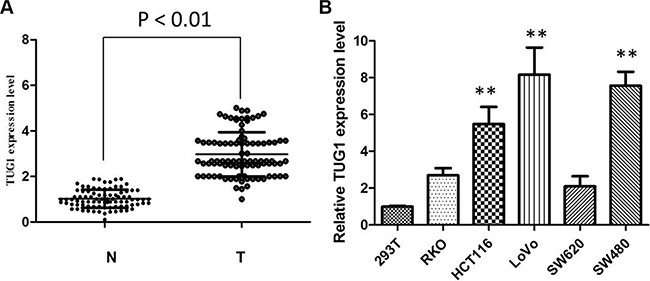
The TUG1 expression levels in CRC tissues and cell lines (**A**) TUG1 was detected in CRC tissues and adjacent normal mucosa tissues by qRT-PCR; (**B**) qRT-PCR showing expression level of TUG1 mRNA in CRC cell lines (**P < 0.01).

We detected TUG1 expression in 88 patients, with paired CRC tissues and corresponding adjacent normal mucosa tissues. It showed that the TUG1 expression was higher in 57 patients (64.77%, 57 of 88) than the adjacent ones (*P* < 0.01; Figure 1A). To confirm the role of TUG1 in CRC, we also performed the qRT-PCR analysis to evaluate the TUG1 expression in a panel of CRC cell lines (HCT116, SW480, LoVo, SW620, and RKO). Figure 1B revealed that LoVo and SW480 cells are of higher TUG1 expression. Thus, LoVo and SW480 cells were used as a model to performed following investigation of TUG1 on cell proliferation, apoptosis, migration and invasion in CRC *in vitro*.

**Figure 2:**
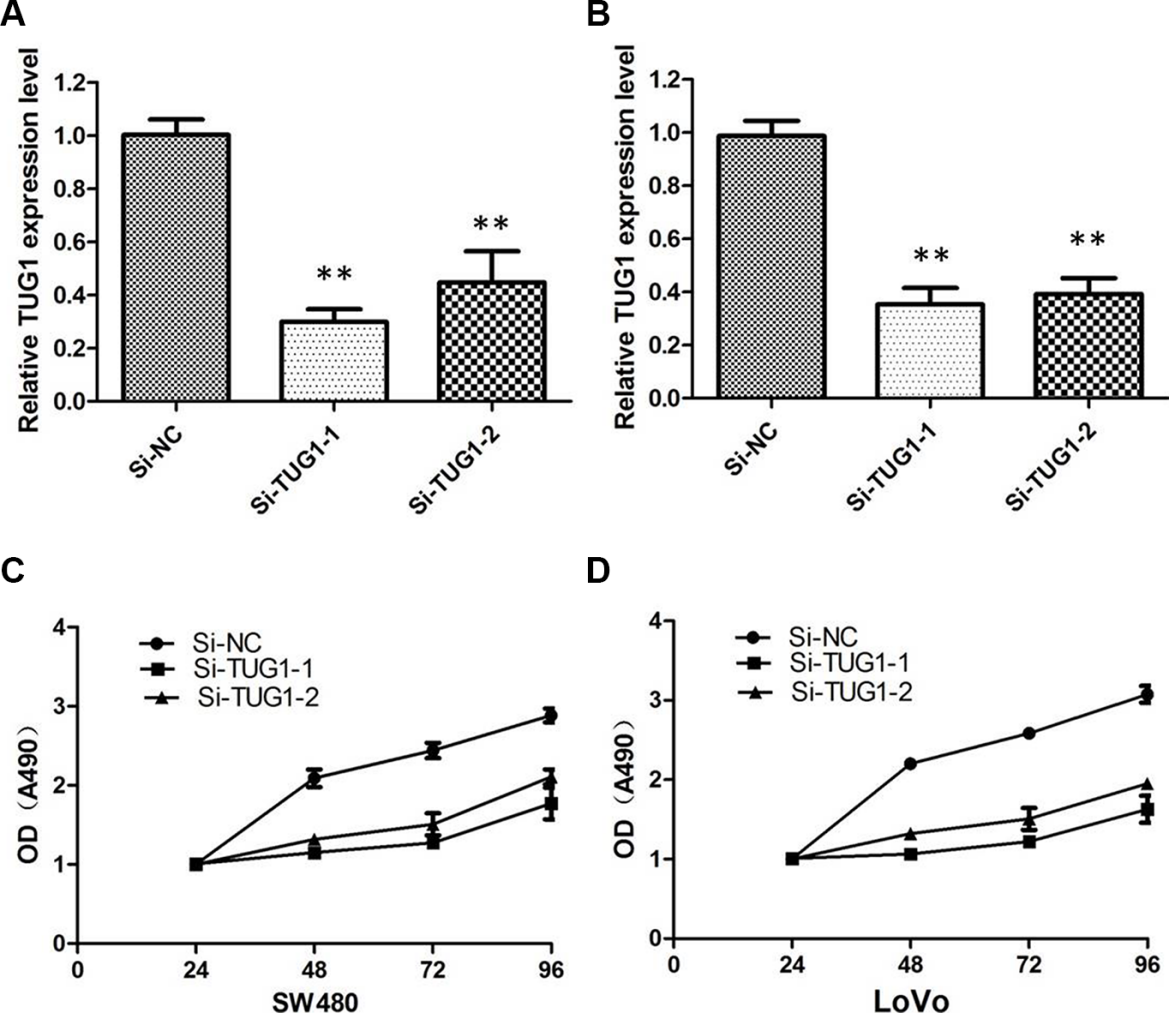
Knockdown efficiency of TUG1-specific siRNA in CRC cells (**A**) qRT-PCR showing the expression of TUG1 mRNA in LoVo/si cells was significantly decreased compared with control cells (**P < 0.01); (**B**) qRT-PCR showing the expression of TUG1 mRNA in SW480/si cells was significantly decreased compared with control cells (**P < 0.01); (**C**) MTT assay showing TUG1 knock down inhibited cell proliferation of LoVo cells; (**D**) S TUG1 knock down inhibited cell proliferation of SW480 cells.

**Figure 3:**
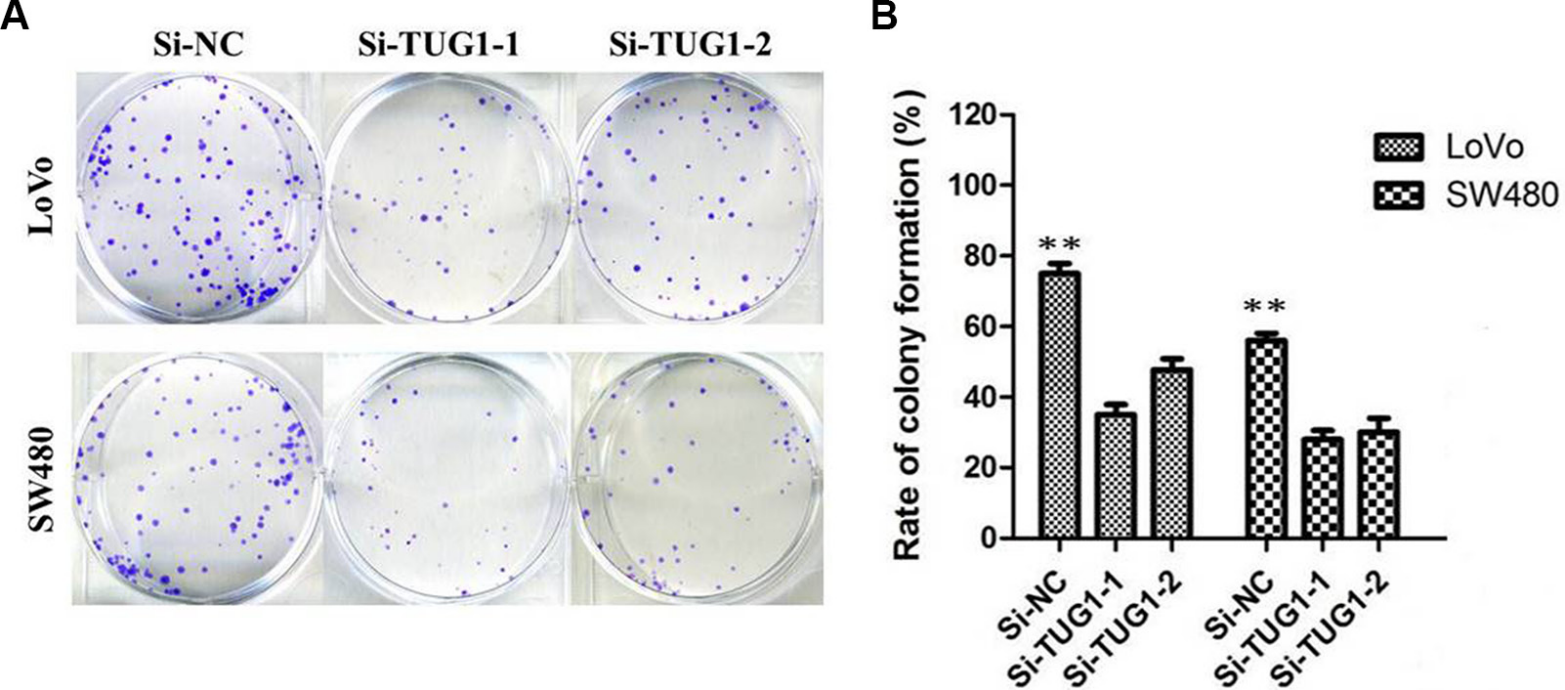
Colony-formation assays showed that silencing of TUG1 significantly increased the colony-forming ability of LoVo and SW480 cells (**P < 0.01)

**Figure 4:**
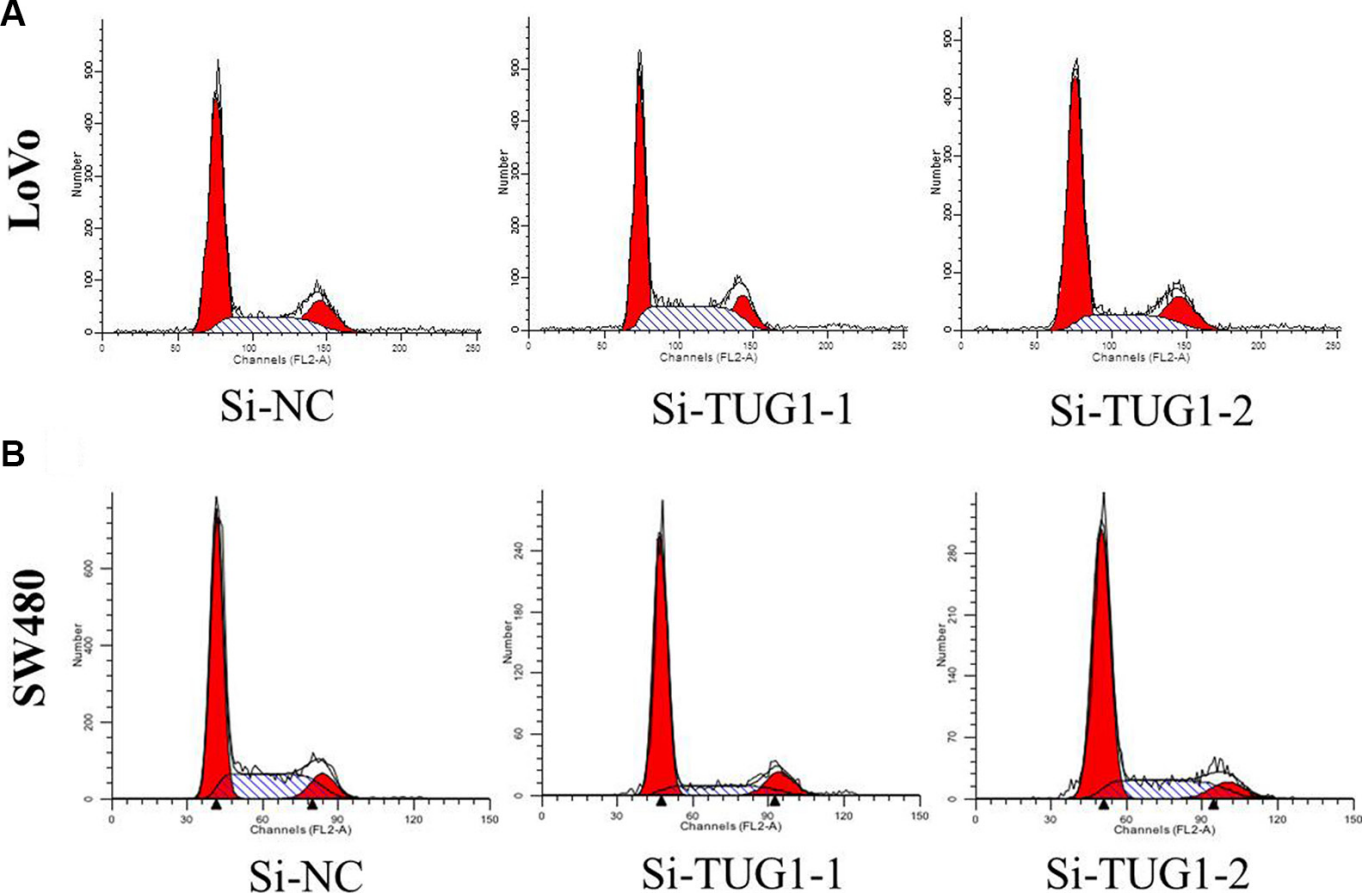
(**A**) LoVo cells transfected with si-TUG1 had cell-cycle arrest at the G1-G0 phase compared with cells transfected with si-NC; (**B**) SW480 cells transfected with si-TUG1 all had cell-cycle arrest at the G1-G0 phase compared with cells transfected with si-NC.

**Figure 5:**
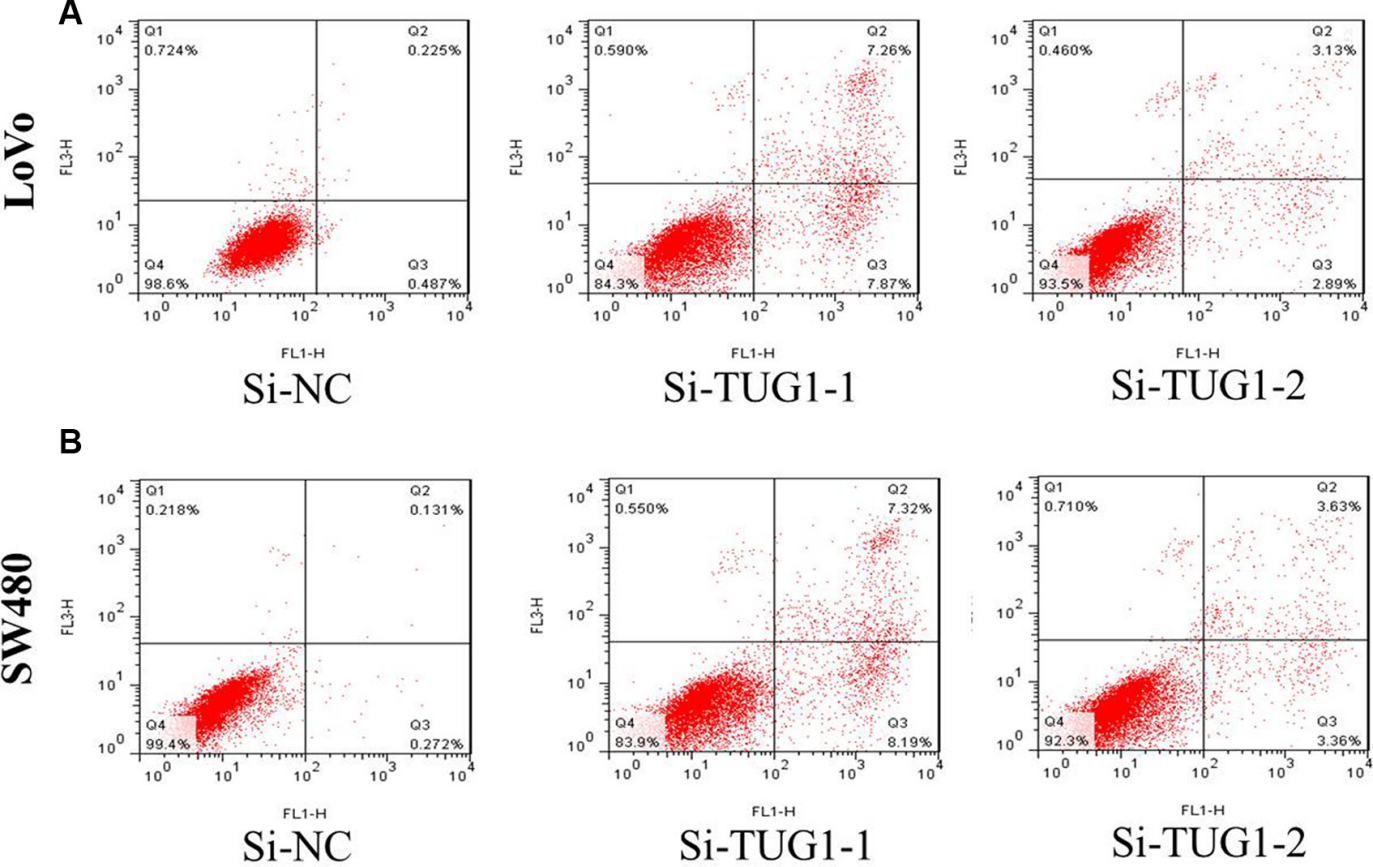
(**A**) the proportion of apoptotic cells following TUG1 siRNA treatment was increased in LoVo cells; (**B**) the proportion of apoptotic cells following TUG1 siRNA treatment was increased in SW480 cells.

Then, we designed two different TUG1 siRNAs to knockdown the TUG1 expression in LoVo and SW480 cells. The qRT-PCR assay was performed at 48 h post-transfection to confirm the silencing efficiency. We could see that TUG1 expression was significantly reduced after transfection with si-TUG1-1# and si-TUG1-2# (Figure 2A and 2B). Then MTT assay showed that TUG1 knockdown significantly weakened LoVo and SW480 cells’ vitality of (Figure 2C and 2D). Consistently, colony formation assays implied that clonogenic ability was significantly decreased after inhibition of TUG1 in LoVo and SW480 cells by si-TUG1-1# and si-TUG1-2# (Figure 3A and 3B). Moreover, flow cytometric analysis implicated that the cell-cycle was significantly arrested at the G1-G0 phase in silencing groups compared with NC (Figure 4A and 4B). Additionally, cell apoptosis was also performed, which revealed a obviously reduced apoptosis in silencing group of both cell lines (Figure 5A and 5B).

**Figure 6:**
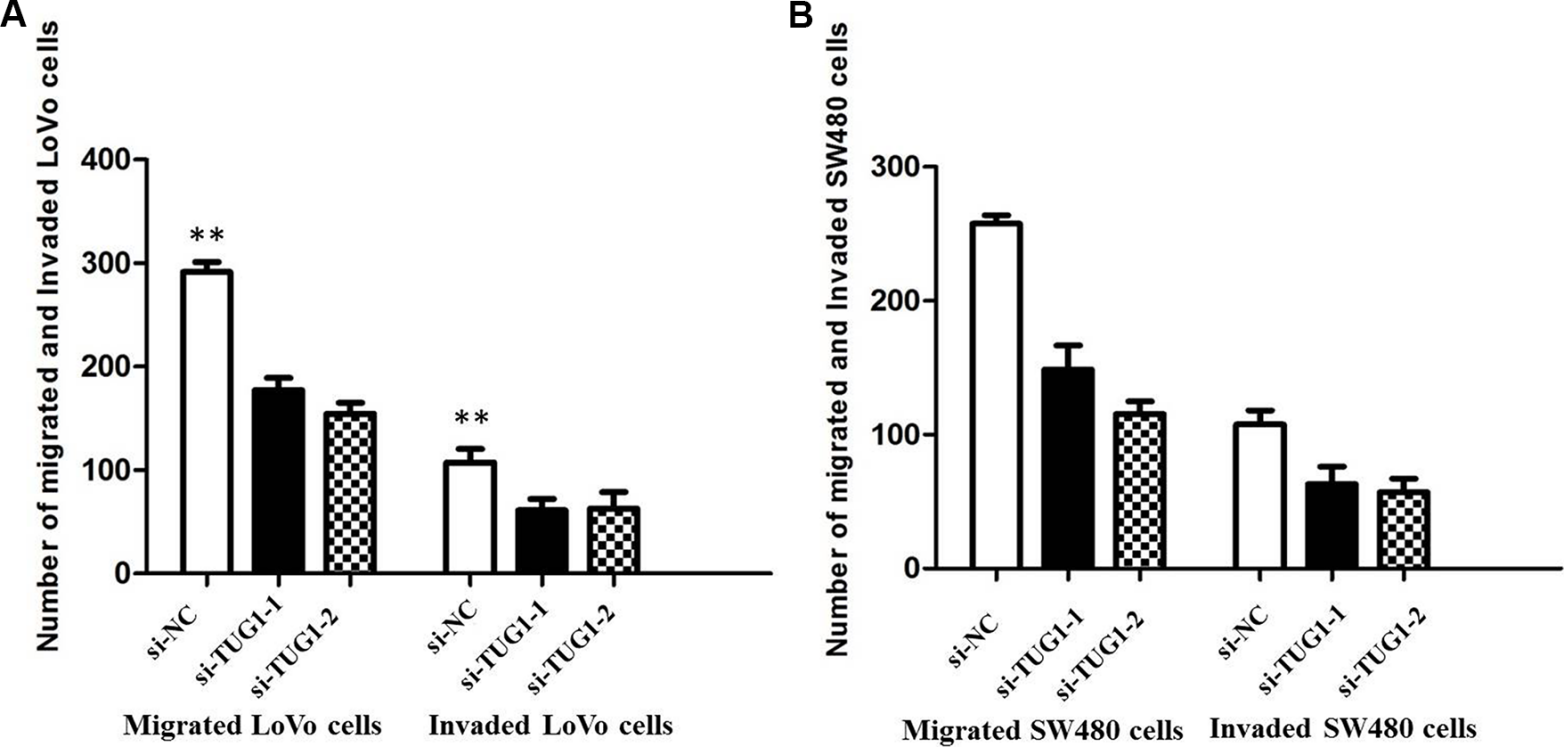
(**A**) Inhibition of Migration and Invasion of LoVo cells by TUG1 siRNA(**P < 0.01); (**B**) Inhibition of Migration and Invasion of SW480 cells by TUG1 siRNA (**P < 0.01).

**Figure 7:**
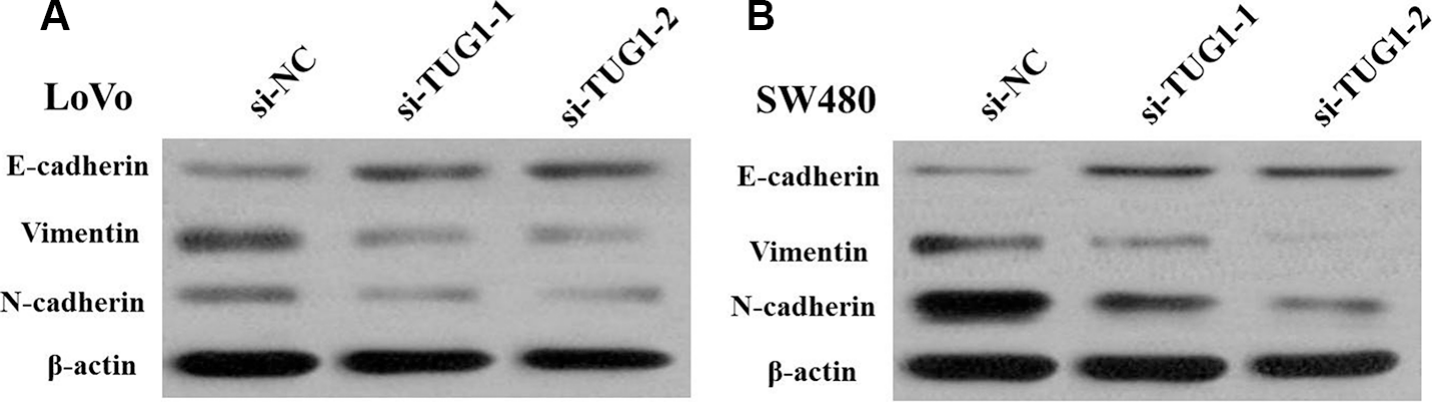
(**A**) Knockdown of TUG1 reverses EMT in LoVo cells; (**B**) Knockdown of TUG1 reverses EMT in SW480 cells.

Besides, the migration and invasion assays demostrated that siRNA treatment significantly impaired migration and invasion capacity in comparison with NC groups (Figure 6A and 6B). we also detected EMT-relavant proteins. As shown in Figure 7, downregulation of TUG1 in LoVo and SW480 cells remarkably increased the E-cadherin expression and meanwhile greatly decreased the expression of Vimentin.

## DISCUSSION

In our study, we detected the TUG1 expression in CRC and further explored functional role and possible mechanism of lncRNA TUG1. Despite of the great progress in early diagnosis, surgical techniques and chemotherapy, the prognosis of patients with CRC is still unsatisfactory. Aberrant expression of lncRNAs has been found associated with CRC progression [13–14]. So, identification of novel biomarkers, especially CRC-associated lncRNAs, is an urgent need for advanced CRC to realise early diagnosis, and work out effective strategy with precise target, improving patients’ prognosis.

Pervasive studies showed that TUG1 is aberrantly expressed and participated in tumor development and progression [15]. In our study, we investigated the TUG1 expression in tumor tissues and matched adjacent normal mucosa tissues from 88 patients with CRC, and a panel of CRC cell lines. We also conducted a series of experiments to explore the role of TUG1 acted in CRC development. It is obviously see that silencing TUG1 inhibited cell proliferation, and impaired migration and invasion ability. EMT is a main mechanism involved in cell migration and invasion. We then, focus on two critical proteins, which are universally believed as markers of EMT, E-cadherin and Vimentin. Interestingly, TUG1 knockdown upregulated E-cadherin expression while downregulated Vimentin expression. These demonstrated that TUG1 affects CRC cell proliferation and metastasis partly through the EMT. This study advances our understanding of the role of TUG1 as a regulator of CRC pathogenesis.

Previous studies indicated that TUG1 was highly expressed in bladder carcinoma, osteosarcoma and esophageal squamous cell carcinoma [16–19]. However, TUG1 has been found to be down-regulated in human glioma, indicating that TUG1 is tissue-specific and may function as oncogene or tumor suppressor in different cancer [20]. First, qRT-PCR is used to investigate the expression of TUG1 in CRC tissues samples and cell lines. We confirmed that TUG1 is highly expressed in most of CRC tissues and all CRC cell lines, compared with normal ones. LoVo and SW480 cells were chosen to be performed the following experiment due to the top two expression of TUG1.

A series of functional analysis was done after silencing TUG1 expression in LoVo and SW480 cells. Knockdown of lnc RNA TUG1 led to proliferation inhibition of LOVO and SW480 cells, concomitant with induction of cell apoptosis and inability to metastasize. Moreover, downregulation of TUG1 significantly induced G0/G1 arrest.

A mount of studies showed that TUG1 promoted cancer cell invasion and radioresistance via EMT [21–22]. In the present study, we identified that TUG1 knockdown could weaken migratory and invasive ability LOVO and SW480 cells by regulating EMT progress. Downregulation of TUG1 in CRC cells remarkably led to upregulation of E-cadherin and downregulation of vimentin.

In summary, we demonstrated that TUG1 significantly contributes to CRC progression. Inhibition of TUG1 could inhibit CRC cell proliferation, migration and EMT. Because of this crucial role TUG1 plays in the progression of CRC, it holds great promise as a potential therapeutic target.

## MATERIALS AND METHODS

### Patients and sample collection

Pairs of fresh CRC tissues and paired adjacent noncancerous tissues were obtained from 88 patients undergoing surgical procedures at First Affiliated Hospital, Sun Yat-sen University, between January 2010 and January 2014. Both tumors and noncancerous tissues were subjected to histological analysis for diagnostic confirmation. The pathological type of each cancer was identified as adenocarcinoma. After resection, all samples were immersed immediately in RNA later solution (Ambion, Austin, Texas) overnight, and stored at −80°C in order to avoid degradation of RNA. Prior to the use of these clinical materials for research purposes, written consents from all patients and approval of the First Affiliated Hospital, Sun Yat-sen University Ethic Review Committees were obtained.

### Cell lines and culture conditions

Five CRC cell lines (HCT116, LoVo, RKO, SW620, and SW480) and 293T cell line were purchased from the Institute of Biochemistry and Cell Biology of the Chinese Academy of Sciences (Shanghai, China). Cells were cultured in RPMI 1640 or DMEM (Gibco, Grand Island, NY, USA) medium supplemented with 10% fetal bovine serum (10% FBS), 100 U/ml penicillin, and 100 mg/ml streptomycin (Gibco) in humidified air at 37°C with 5% CO_2._

### RNA extraction and qRT-PCR analyses

RNA was extracted using TRIzol reagent (Invitrogen) and qRT-PCR was performed for TUG1 using GAPDH as an internal control. Total RNA was then converted to cDNA by reverse transcription using oligodT primers and SuperScript II reverse transcriptase (Invitrogen). For qRT-PCR, three replicates of each sample were amplified in a 20 μL reaction mixture containing SYBR Green reaction mix (Qiagen, Germany) and 0.5 mM of primer, and analyzed using a Roche Light-Cycler (Roche, Basel, Switzerland). The PCR primers for TUG1 or GAPDH were as follows: TUG1 forward, 5′-CTGAAGAAAGGCAACATC-3′′ and reverse, 5′-GTAGGCTACTACAGGATTTG-3′; GAPDH forward, 5′-AGCCACATCGCTCAGACAC-3′ and reverse, 5′-GCCCAATACGACCAAATCC-3′. The relative gene expression in cells was determined using the comparative delta-delta CT method (2-ΔΔCt) and the fold change in gene expression of tissues was calculated using the standard ΔΔCT method.

### Transfection

Small interfering RNA (siRNA) against TUG1 (si-TUG1) and nonspecific control siRNA were synthesized (Carlsbad, California, USA) and transfected into cells using Lipofectamine 2000 (Invitrogen, USA). The sequences of the three designed TUG1 siRNAs were as follows: si-TUG1 1#, CAGUCCUGGUGAUUUAGACAGUCUU; si-TUG1 2#, CCCAGAAGUUGUAAGUUCACCUUGA. Cells were harvested after 48 h for qRTPCR and western blot analyses.

### Cell proliferation and clonogenic assay

Cell proliferation assay was carried out using the MTT kit according to the manufacturer's instruction (Roche, Basel, Switzerland). Briefly, after transfection for 48 h, 3,000 cells per well were allowed to grow in 96-well plates with five replicate wells. After 6 h of culture, as well as at 24, 48, 72 and 96 h after atarting the culture, the cells were treated with 100 μg MTT by adding it to the medium. The cells were incubated at 37°C for another 4 h, then the medium was removed, and DMSO was added for 10 min to lyse the cells. Finally, the absorbance at a wave length of 490 nm was determined using a microplate reader. For clonogenic assay, 500 cells were plated in each well of a six-well plate. When there was visible colony by naked eye, cells were fixed with methanol and stained with 0.1% crystal violet (Sigma, USA). Colonies were then counted.

### Flow cytometric analysis of apoptosis and cell cycle

Transfected cells were harvested after transfection by trypsinization. After the double staining with fluorescein isothiocyanate (FITC)-Annexin V and propidium iodide was done by the FITC Annexin V Apoptosis Detection Kit (BD Biosciences) according to the manufacturer's recommendations, the cells were analyzed with a flow cytometry (FACScan; BD Biosciences) equipped with a Cell Quest software (BD Biosciences). Cells were discriminated into viable cells, dead cells, early apoptotic cells, and apoptotic cells and then the relative ratio of early apoptotic cells were compared with control transfection from each experiment. Cells for cell cycle analysis were stained with propidium oxide by the CycleTEST PLUS DNA Reagent Kit (BD Biosciences) following the protocol and analyzed by FACScan.

### Migration and invasion assay

Cell migration or invasion assays were performed using a 24-well Transwell chamber (Costar, Massachusetts, USA) with or without Matrigel coating. After 48 h, cells (4 × 10^4)^ were detached and seeded into the upper chamber of an 8-μm pore size insert in the 24-well plate and cultured for another 12 h. The cells were allowed to migrate or invade the bottom chamber containing 15% FBS. The nonmigratory cells on the surface of the upper membrane were removed with a cotton tip, and the migratory or invasive cells attached to the lower membrane surface were fixed with 4% paraformaldehyde and stained with crystal violet. The number of migratory and invasive cells was counted in five randomly selected high-power fields under a microscope. The presented data represent three individual wells.

### Western blot analysis

Cells were lysed in lysis bufferin the presence of Aprotinin, Leupeptin, Phenylmethanesulfonyl fluoride (PMSF) (Sigma) and phosphatase inhibitor cocktails II and III (Sigma). Proteins were quantied by Bradford method. Then, 50 mg of total protein extracts was fractionated by 10% sodium dodecyl sulfate-polyacrylamide gel electrophoresis and transferred to polyvinylidene diuoride membranes (GE Healthcare, Piscataway, NJ, USA). The membrane was incubated with the following primary antibodies: anti E-cadherin, anti-N-cadherin, anti-Vimentin (Santa Cruz Bio-technology, Santa Cruz, CA, USA), and anti-β-actin antibody (Cell Signaling Technology). Binding of the primary antibody was detected using an enhanced chemiluminescence kit (ECL Amersham).

### Statistical analysis

STATA 9.2 and Graph Pad prism software were used for data analysis. Data of cell samples are presented as mean ± SEM for three times every test, that are analyzed by double-sided Student's *t*-test. Results were considered statistically significant at *P* < 0.05.
